# In Vitro Assessment of Anthelmintic Activities of* Rauwolfia vomitoria* (Apocynaceae) Stem Bark and Roots against Parasitic Stages of* Schistosoma mansoni* and Cytotoxic Study

**DOI:** 10.1155/2017/2583969

**Published:** 2017-02-28

**Authors:** Emmanuel Mouafo Tekwu, Kwabena Mante Bosompem, William Kofi Anyan, Regina Appiah-Opong, Kofi Baffour-Awuah Owusu, Mabel Deladem Tettey, Felicia Amanfo Kissi, Alfred Ampomah Appiah, Veronique Penlap Beng, Alexander Kwadwo Nyarko

**Affiliations:** ^1^Laboratory for Tuberculosis Research and Pharmacology, Biotechnology Centre, Nkolbisson, University of Yaoundé 1, Yaoundé, Cameroon; ^2^Noguchi Memorial Institute for Medical Research (NMIMR), College of Health Sciences, University of Ghana, PO Box LG581 Legon, Accra, Ghana; ^3^Centre for Plant Medicine Research (CPMR), Akwapim, Mampong, Ghana; ^4^School of Pharmacy, College of Health Sciences, University of Ghana, Accra, Ghana

## Abstract

Schistosomiasis is a Neglected Tropical Diseases which can be prevented with mass deworming chemotherapy. The reliance on a single drug, praziquantel, is a motivation for the search of novel antischistosomal compounds. This study investigated the anthelmintic activity of the stem bark and roots of* Rauwolfia vomitoria* against two life stages of* Schistosoma mansoni*. Both plant parts were found to be active against cercariae and adult worms. Within 2 h of exposure all cercariae were killed at a concentration range of 62.5–1000 *µ*g/mL and 250–1000 *µ*g/mL of* R. vomitoria* stem bark and roots, respectively. The LC_50_ values determined for the stem bark after 1 and 2 h of exposure were 207.4 and 61.18 *µ*g/mL, respectively. All adult worms exposed to the concentrations range of 250–1000 *µ*g/mL for both plant parts died within 120 h of incubation. The cytotoxic effects against HepG2 and Chang liver cell assessed using MTT assay method indicated that both plant extracts which were inhibitory to the proliferation of cell lines with IC_50_ > 20 *μ*g/mL appear to be safe. This report provides the first evidence of in vitro schistosomicidal potency of* R. vomitoria* with the stem bark being moderately, but relatively, more active and selective against schistosome parasites. This suggests the presence of promising medicinal constituent(s).

## 1. Introduction

Schistosomiasis (or Bilharzia) is known as one of the most prevalent tropical diseases worldwide. It is ranked as the second-most neglected tropical disease in terms of the number of deaths it causes [1]. Schistosomiasis is responsible for more than 200,000 human deaths per year in sub-Saharan Africa alone [2]. The disease is better known for its chronicity and debilitating morbidity which results in high costs in public health and economic productivity in developing countries [3].

Despite the public health importance of schistosomiasis and the risk that the disease might further spread and intensify, schistosomiasis control programs in endemic countries are based mainly on chemotherapy [4]. As it is today for some neglected tropical diseases such as trypanosomiasis and leishmaniasis, the treatment of schistosomiasis was almost as difficult and toxic until the 1970s when praziquantel (PZQ) was discovered [5, 6]. In 1988, PZQ was brought to the market [5] and it is so far the only drug available and recommended by World Health Organization (WHO) for the treatment and control of schistosomiasis [6]. This drug is safe and effective against all* Schistosoma* species [7] and has been used for the last 30 years. PZQ is not free of problems, although it is safe and well tolerated. For instance, the massive and exclusive use for many decades as a single drug has obviously raised legitimate fears that PZQ-resistant schistosomes may sooner or later appear [7]. Furthermore, PZQ acts against adult schistosome worms, but it is inefficient against the younger stages of schistosomes, like schistosomula, preadults, and juvenile adults. As a consequence, repetition of treatment is sometimes necessary to kill those parasites that have since matured.

Having a single drug to treat a disease that affects millions of people in different geographical area is a real concern. Therefore, it is imperative to develop new effective and safe antischistosomal drugs. The growing need for the development of novel drugs against schistosomiasis mainly from natural sources has, in recent years, led the scientific community to intensify the search for potential schistosomicidal agents. Natural products, mainly plants, have been the source of medicines for thousands of years [8]. Higher plants have been used as natural sources for the discovery of new drug leads, since the scientific evaluation of medicinal plants used in the preparation of folk remedies has provided modern medicine with effective pharmaceuticals for the treatment of diseases caused by parasites. For instance, artemisinin, quinine, and licochalcone A are examples for plant-derived products [9]. Many other natural products of diverse molecular structure have shown antiparasitic potency in the laboratory and represent interesting lead structures for the development of new and urgently needed antiparasitics [9]. The discovery of pure compounds as active principles in plants was first described at the beginning of the 19th century [9]. As a result, several extracts or natural compounds from plants with promising antischistosomal properties have been identified [9–15]. In this context, natural products and natural product-derived compounds are gaining prominence as possible sources of new drugs in the control and treatment of schistosomiasis. The efficacy of these new compounds against schistosome is defined using different strategies such as curative strategies, by killing the adult worm; prophylactic strategies, by killing cercariae and schistosomula; suppressive strategies, by inhibiting worm egg-laying. Thus, several parameters, such as motor activity, morphological/tegumental changes, and oviposition, are often evaluated as indicators of biological activity and toxicity in studies with schistosome species [16].

*Rauwolfia vomitoria* is a tropical shrub which belongs to the family of Apocynaceae. In Africa, the herbal preparations have been made from various parts of this plant. However, the root, root bark, and bark of stem of this plant have been used extensively, particularly for their aphrodisiac, antipsoric, abortive, insecticidal, dysenteric, anthelmintic, astringent, cardiotonic, diaphoretic, emetic, expectorant, haemostatic, hypotensive, vulnerary, and febrifugic properties [17]. They are also used in traditional medicine to treat a variety of illnesses such as fever, general weakness, gastrointestinal diseases, liver diseases, psychosis, pain, and cancers [18]. The ethanolic extract of* R. vomitoria* has been shown to contain alkaloids, tannins, saponins, flavonoids, steroids, terpenoids, and cardiac glycosides [19]. Various alkaloids were isolated from this plant [18, 20] and many of these alkaloids have been isolated from the stem and root. These have shown biological activities such as antimalarial, anticancer, and schistosomicidal properties. The alkaloids from* R. vomitoria* were found to have anticancer activity [18, 21]. The extract of* R. vomitoria* has been used as traditional medicine for over 2000 years in Africa for the treatment of hypertension and mental disorders and recent studies have confirmed its effectiveness as antipsychotic, antihypertensive, and anti-inflammatory agent and for improving blood chemistry. In Ghana, root specimens of* R. vomitoria* are used as emetic and purgative and to treat parasitic skin diseases [22] and jaundice and gastrointestinal disorders [23]. It was reported that the bark of* R. vomitoria* can be used against such parasites as lice and scabies. Attah et al. (2013) showed that roots of* R. vomitoria* possess antifilarial properties against* Onchocerca volvulus* [24]. In this study, we evaluated the root and stem bark of* R. vomitoria* as an important but not yet known aspect of the schistosomicidal effect on two different life stages of* S. mansoni*. Both plant extracts were subsequently screened against mammalian cells: human hepatocarcinoma cell lines (HepG2) and Chang liver cell lines to check for cytotoxicity.

## 2. Material and Methods

### 2.1. Chemicals

Ethanol, 3-(4,5-dimethyl thiazol-2-yl)-5-diphenyl tetrazolium bromide (MTT), fetal bovine serum (FBS), Phosphate Buffered Saline (PBS), Dulbecco's Modified Eagle's Medium (DMEM), antibiotics (penicillin and streptomycin), RPMI 1640, trypsin, dimethyl sulfoxide (DMSO), hydrochloric acid, and isopropanol were obtained from Sigma Aldrich Co., USA.

### 2.2. Collection of Plant Materials

The stem bark and roots of* R. vomitoria* were collected in Mampong, Ghana, in 2016 and authenticated by the Plant Development Department (PDD) of the Centre for Plant Medicine Research (CPMR), and voucher specimens were deposited at the Herbarium of the PDD (voucher specimen numbers 3827–3829).

### 2.3. Preparation of Plant Extracts

The plant extracts were prepared as described previously [19] with some modifications. Briefly, the stem bark and the roots of the collected plants were air dried at room temperature (25–27°C) and pulverized by milling. Each pulverized material was subjected to extraction using absolute ethanol (99%). One (1) kg of the pulverized plant parts was macerated in 2 L of ethanol solvent in glass containers for 72 h at room temperature after which they were filtered with Whatman Number 1. The filtrate of each plant extract was evaporated at 55°C under reduced pressure using rotary evaporator (Buchi Rotavapor, R 200) to obtain a crude extract. The total extraction yield for each extract expressed as a percentage was estimated to be 11.2% and 15.1%, respectively, for* R. vomitoria* stem bark and root. The plant extracts were stored at −20°C until used.

### 2.4. In Vitro Studies with* S. mansoni*

#### 2.4.1. Stock and Working Plant Extract Solutions

In vitro studies were conducted on two different life stages of* S. mansoni*, cercariae and adult worms. Stock solutions of extracts were prepared in advance at a concentration of 100 mg/mL in 100% dimethyl sulfoxide (DMSO, Merck, Germany). Stock solutions were aliquoted and kept at −20°C. For bioassay, working solutions were freshly prepared by diluting stock solutions to 10 mg/mL using distilled water for cercariacidal activity and RPMI 1640 for drug sensitivity assay with adult schistosomes. The maximum final concentration of DMSO in all assays was ≤1%. Since DMSO is known to be cytotoxic at higher concentrations, we tested DMSO at concentrations of 1% and found no damaging effects on any of the life stages of the parasite. And, for each experiment, 1% DMSO was used as a solvent control.

#### 2.4.2. Animals

An African strain of* S. mansoni, *from Ghana, was maintained in the laboratory using* Biomphalaria pfeifferi* snails and female ICR outbred mice. All animal studies were conducted at the Animal Experimentation Department of the Noguchi Memorial Institute for Medical Research (NMIMR), following NMIMR Institutional Animal Care and Use Committee (NIACUC) regulations on animal welfare.

Four-to-five-week-old ICR mice were obtained from the Centre for Plant Medicine Research (CPMR) (Mampong-Akwapim, Ghana). All animals were allowed to adapt for 1 week under controlled conditions (temperature, ca. 22°C; humidity, ca. 50%; 12 h light and 12 h darkness cycle; free access to rodent diet and water) before initiation of experiments. Mice were tail-infected with 150* S. mansoni* cercariae and sacrificed 12 weeks after infection.

The snails* B. pfeifferi,* which are the schistosomiasis intermediate host snails for* S. mansoni, *were collected from endemic areas in their natural habitats from Tomefa along the Weija River in Ghana. The snails were transported to the Snail Laboratory at the Department of Parasitology, NMIMR, for maintenance. They were examined for cercariae shedding and kept in plastic aquarium as 50 snails per each aquarium containing dechlorinated tap water at room temperature (25°C).

#### 2.4.3. Preparation of Cercarial Suspension

Schistosome cercariae were obtained from experimentally infected* B. pfeifferi *snails. Briefly, five laboratory infected snails for about 4 weeks and known to be shedding cercariae were placed into a test tube containing 2 mL of distilled water and allowed to shed cercariae by exposing them to artificial light at 28°C ± 1 for 2 h. The number of cercariae in 50 *μ*L was counted microscopically in triplicate samples and the average count used for anticercariae evaluation at an average of 20 cercariae per well was then estimated. For the experimental mice infection, each animal was exposed by the tail to a suspension of approximately 150-cercariae in glass test tubes for 60 min.

#### 2.4.4. Preparation of Adults* S. mansoni* Worms

Twelve (12) weeks after infection, ICR mice were euthanized with chloroform and dissected. All adult worms were recovered from the hepatic portal and mesenteric veins by perfusion with citrate saline (0.85% sodium chloride; 1.5% sodium citrate) as previously described [20]. Schistosomes were washed in RPMI 1640 culture medium supplemented with 10% fetal bovine serum (FBS), 100 U/mL penicillin, and 100 *μ*g/mL streptomycin (Sigma Aldrich, USA) and counted. The worms were transferred into RPMI 1640 culture medium, modified with HEPES 20 mM and L-Glutamine, without Sodium Bicarbonate.

#### 2.4.5. In Vitro Cercariacidal Activity Test

A series of crude plant extract concentrations (31.25, 62.5, 125, 250, 500, and 1000 *μ*g/mL) were freshly prepared in a 24-microtiter well plate (Costar) and analyzed alongside with the positive control (Artesunate 10 *μ*g/mL). An average number of 20 freshly shed cercariae were transferred into each well plate (Costar) using micropipette. The same number of cercariae was placed in a well containing 1% DMSO as negative control. All experiments were carried out in duplicate and were repeated. Mobility and viability of the* Schistosoma* infectious stage (cercariae) were observed for 2 h 30 min at 30 min intervals since infectivity of cercariae is known to be rapidly lost after 12 h [25]. Unaffected free swimming larvae, immobile, and dead cercariae at the bottom of the wells were observed at 4x magnification with an inverted microscope (Olympus CK 300). Survival and mortality at a successive interval of 15, 30, 60, 90, 120, and 150 min were recorded. Cercariae were presumed dead when they stopped movement and sank down and their tail were detached [26]. The LC_50_ values of the plant extract on schistosome cercariae were determined at 1 h and 2 h. The minimal lethal concentration (MLC), which is the minimum concentration needed to kill all cercariae, and the minimal effective concentration (MEC), which is the minimum concentration needed to observe any change in viability or morphology of cercariae, were determined after 2 h.

#### 2.4.6. Drug Sensitivity Assay with Adult Schistosomes

For the in vitro drug sensitivity assay with adult schistosome worms, RPMI 1640 culture medium, modified with HEPES 20 mM and L-Glutamine, without Sodium Bicarbonate and supplemented with 10% FBS, 1% penicillin/streptomycin was pipetted (2 mL/well) into flat-bottom 24-well plates. The worms were distributed one pair of adult worms per well and incubated at 37°C in a 5% CO_2_ atmosphere for 2 h to allow for adaptation before addition of various concentrations of the plant extracts. Final concentrations of each crude plant extract are 250, 500, and 1000 *μ*g/mL in a final volume of 2 mL per well. Adult worms incubated with only medium and medium with 1% DMSO (the highest concentration of drug solvent used) served as negative and solvent control, respectively. For positive controls, adult worms were incubated with 10 *μ*g/mL PZQ. After 2 h of incubation, the adult worms in the individual wells were observed under an inverted microscope for viability. The effect of the plant extracts on the worms was assessed as described previously [1, 13, 14, 27–29]. The parasites were kept for 120 h and monitored every 24 h to evaluate their general condition with emphasis on changes in worm motor activity (motility), morphological/tegumental changes, changes in pairing, and death of worms at magnification 4x under an inverted microscope (Olympus CK 300). Death was assessed with no movement observed for at least 2 min of examination and no movement at the other observation time-points. Phenotypic changes were recorded manually as previously reported, WHO-TDR [30] and UCSF Sandler Center [31]. Briefly, the changes were converted into a “severity score” using a viability scale ranging from 0 (severely compromised) to 3 (no effect): (0 = all worms dead, 1 = minimal activity (severe reduction in motility), severe morphological/tegumental changes, 2 = slowed activity (reduced motility), first morphological/tegumental changes, and 3 = totally vital, normally active, no morphological changes) based on standard procedures for compound screening. All experiments were carried out in triplicate and were repeated at least two times.

### 2.5. Cytotoxicity of the Prepared Extracts

The two plant parts showing activity against the two parasite stages were tested in concentration response (CR) assays against two cell lines: HepG2 (human hepatocarcinoma) cell line was procured from American Type Culture Collection (ATCC) and Chang liver was obtained from the European Collection of Authenticated Cell Cultures (ECACC). Stock cells HepG2 and Chang liver were, respectively, cultured in DMEM and RPMI1640. Each medium was supplemented with 10% FBS and 1% penicillin-streptomycin and cultures were then incubated at 37°C under 5% CO_2_ in fully humidified conditions until 80% confluence. The stock cultures were grown in 25 cm^2^ culture flasks and cells were detached from the surface of the culture flask with 0.25% trypsin solution. All experiments were carried out in flat-bottom 96-well microtiter plates (Corning Incorporated, USA).

Fresh stock solutions of the plant extracts were made up with DMEM (for HepG2 cell) and RPMI1640 (for Chang liver cell) supplemented with 10% FBS and 1% penicillin-streptomycin to obtain 10 mg/mL concentration and sterilized by filtration. Serial twofold dilutions were prepared from the stock. The monolayer cell culture was trypsinized and the cell count was adjusted to 1.0 × 10^5^ cells/mL using DMEM or RPMI1640 accordingly containing 10% FBS and 1% penicillin-streptomycin. To each well of the 96-well microtiter plates, 100 *μ*L of the diluted cell suspension at a density of approximately 100,000 cells was plated. After 24 h of incubation, when a partial monolayer was formed, cells were treated for another 72 h with various concentrations of each of the plant extracts (62.5–1000 *μ*g/mL) and curcumin (2.30–36.84 *μ*M) as positive control. Subsequently, 20 *μ*L of 2.5 mg/mL MTT in PBS was added to each well and the cells were incubated for another 4 h. The precipitated MTT-formazan product was dissolved in 100 *μ*L of 0.04 N HCl-isopropanol in the dark and at room temperature overnight. The amount of formazan formed was measured at a wavelength of 570 nm using a microplate reader (TECAN Infinite M200 Pro Plate Reader, Austria). Cytotoxicity was calculated as the percentage of live cells relative to the control culture using the following formula:

(1) $$\begin{array}{c} \%\;\;cell\;\;viability\;\left(\;CV\;\right)=100\times \frac{Absorbance\;\;of\;\;treated\;\;cells-Absorbance\;\;of\;\;drug\;\;color\;\;control}{Absorbance\;\;of\;\;untreated\;\;cells-Absorbance\;\;of\;\;blank}. \end{array}$$

The concentration of test drug needed to inhibit cell growth by 50% (IC_50_) values is generated from the dose-response curves for each cell line.

### 2.6. Selectivity Index (SI)

In the present study, the degree of selectivity of each ethanol plant extract is expressed as the ratio of the IC_50_ obtained for the cell line to the LC_50_ for* S. mansoni* cercariae.

(2) Selectivity  Index SI=IC50  in  μg/mL  of  extract  in  Cell  linesLC50  in  μg/mL  of  the  same  extract  in  S.  mansoni  cercariae.

### 2.7. Statistical Analysis

Graph drawing and statistical analysis were performed using GraphPad Software (version 7.00). The data were expressed as means ± SD, and Student's *t*-test was used to determine the significance of differences between mean values. A *p* value of less than 0.05 was considered statistically significant.

## 3. Results

### 3.1. In Vitro Studies of Cercariae: Inverted Microscopic Evaluations

**Figure 1:**
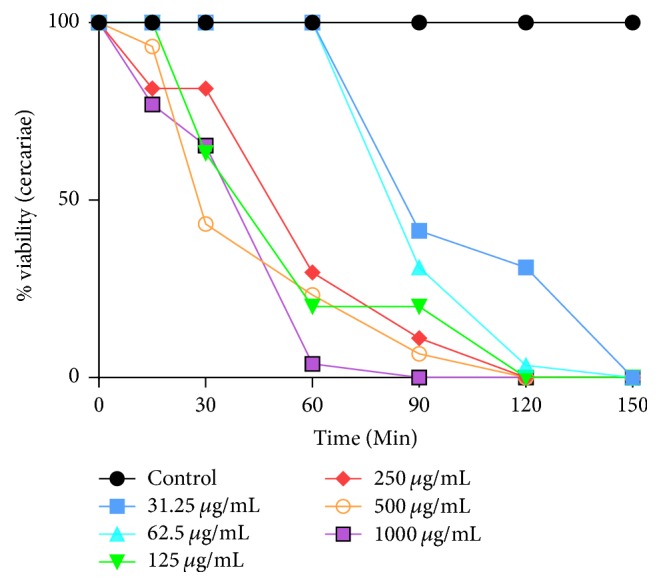
Effect of different concentrations of* R. vomitoria* stem bark on the viability of* Schistosoma mansoni* cercariae.

**Figure 2:**
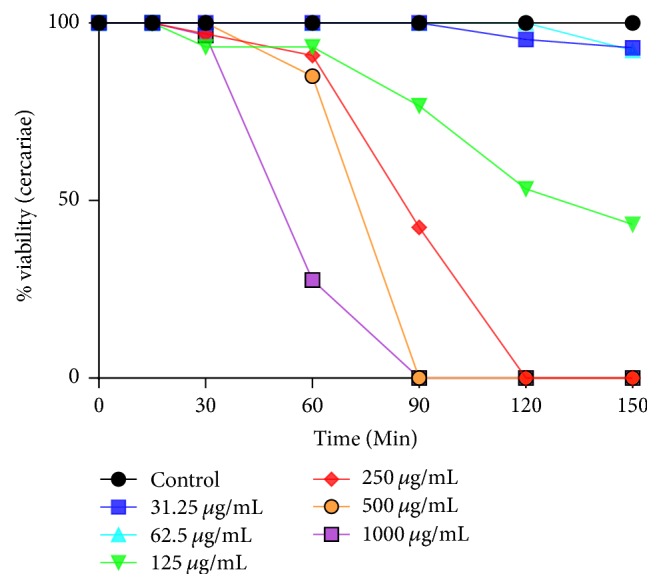
Effect of different concentrations of* R. vomitoria* roots on the viability of* Schistosoma mansoni* cercariae.

*R. vomitoria* stem bark and roots showed varying cercaricidal potency against* S. mansoni* cercariae and this activity was more pronounced at the higher concentration of the extract. The effect of incubation with different concentrations of* R. vomitoria *roots and stem bark extracts on the viability of cercariae for up to 2 h and 30 min is depicted in Figures 1 and 2. The exposure of* S. mansoni* cercariae to the ethanol extract of* R. vomitoria* for 15, 30, 60, 90, 120, and 150 minutes showed an increase in the mortality rate of cercariae. In the absence of the plant extract, cercariae showed normal viability without any morphological changes (tail loss) for up to 2 h.

In this study, anticercarial activity was defined as an LC_50_ value of <1000 *μ*g/mL. Following incubation with* R. vomitoria* stem bark at concentrations of 125–1000 *μ*g/mL, cercariae viability decreased significantly within 1 h. At the highest concentration (1000 *μ*g/mL), there was complete mortality within 90 min (Figure 1), and no movement was observed between 60 and 90 min after incubation. At 1 h, cercariae incubated with 31.25 *μ*g/mL of* R. vomitoria* stem bark showed slowed movements while cercariae incubated with 62.5–500 *μ*g/mL showed severe reduction in activity. However, 2 h after incubation with 62.5, 125, 250, and 500 *μ*g/mL of* R. vomitoria* stem bark there was 100% mortality (Figure 1). All cercariae incubated with 31.25 *μ*g/mL showed 100% mortality at the 2 h and 30 min time-point. Hence, the MLC and MEC of* R. vomitoria* stem bark on cercariae determined after 2 h were 62.5 and 31.25 *μ*g/mL, respectively.

On the other hand,* R. vomitoria *root was less effective than the stem bark. No effect was observed on cercariae at the lower concentrations of 31.25 and 62.5 *μ*g/mL at the 90 min time-point. However, all cercariae died at the higher concentrations (500 and 1000 *μ*g/mL) in 90 min, while, at 250 *μ*g/mL, complete mortality occurred within 2 h (Figure 2). At the 2 h time-point, cercariae incubated with 31.25–62.5 *μ*g/mL of* R. vomitoria* root still showed normal movements compared to controls, while, at 125 *μ*g/mL, cercariae showed reduced activity with tail loss. Hence, the MLC and MEC of* R. vomitoria* root on cercariae determined after 2 h were 250 and 125 *μ*g/mL, respectively.

The LC_50_ values were determined after 1 h and 2 h of exposure to the plant extracts (Table 1). The LC_50_ values of* S. mansoni* cercariae using the* R. vomitoria* stem bark were significantly different (*p* < 0.05) for exposure periods of 1 h and 2 h, respectively. These values were generally lower than those obtained for the same exposure periods to the* R. vomitoria* roots (Table 1). None of the cercariae in the control group died or showed significant behavioral changes within 2 h of exposure. The* R. vomitoria* stem bark extract showed more pronounced cercaricidal potency than the* R. vomitoria *roots.

### 3.2. In Vitro Analysis of Adult* S. mansoni* Worms

We analyzed the schistosomicidal activity of ethanol extracts from two parts of* R. vomitoria* (stem bark and roots) against* S. mansoni* adult worms and the activity was defined as an inhibition value <1000 *μ*g/mL. The differential schistosomicidal activity of* R. vomitoria *extracts was supported by the microscopic observation of* S. mansoni* adult worms incubated with the two plant extracts at a concentration range of 250–1000 *μ*g/mL to examine separation of coupled pairs, decrease in motility, morphological changes, and mortality using a viability scale ranging from 0 to 3. The dose-response relationships of* R. vomitoria* on adult schistosomes for up to 120 h are given in Table 2. Control female and male schistosomes showed normal viability within 48 h and reduced motility from 72 h and remained viable for up to 120 h. After 24 h of incubation schistosomes displayed reduced motility to* R. vomitoria* stem bark (Table 2) and severe reduced motility to* R. vomitoria* root (Table 2) accompanied with separation of paired worms. All adult worms exposed to 250 *μ*g/mL of* R. vomitoria* root and stem bark were dead within 120 h of incubation (Table 2). In the presence of the plant extract, it was observed that females were relatively more active than males within 48 h, and the worm teguments appeared darkened and opaque.

### 3.3. Cytotoxicity of* R. vomitoria* Stem Bark and Roots

Based on the activity against the cercariae and* S. mansoni* adult worms, in the present study, the in vitro cytotoxicity of both stem bark and root ethanol extracts of* R. vomitoria *were evaluated at different concentrations (62.5–1000 *μ*g/mL) against human hepatocarcinoma HepG2 and normal Chang liver cell lines. The cytotoxicity results are presented in terms of percent viability of cells proliferation (Table 3).

**Figure 3:**
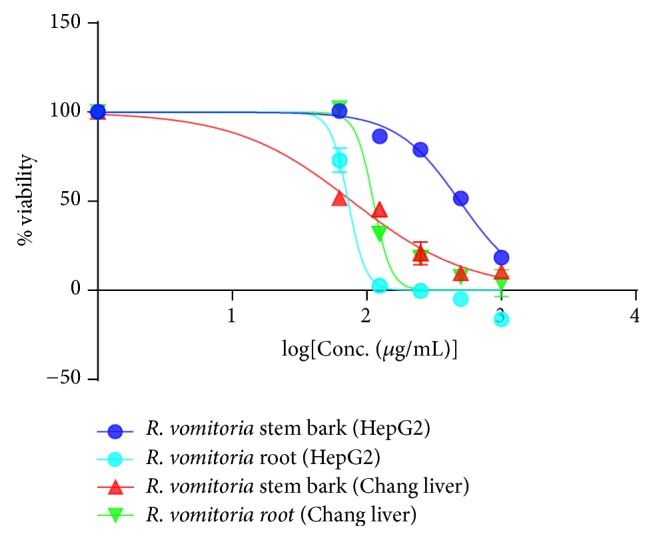
Dose-response curves on the effects of* R. vomitoria* on human HepG2 and Chang liver cells.

The percent cytotoxicity of ethanol extract of* R. vomitoria *was found to be dose dependent and increases with increased concentrations (Figure 3). The dose-response curve (Figure 3) on the effects of* R. vomitoria* on human HepG2 and Chang liver cells was used to define the IC_50_ here presented in Table 4. Considering the cytotoxicity alone, both plant extracts were not strongly cytotoxic against HepG2 and Chang liver cell lines with IC_50_ greater than 20 *μ*g/mL.

Looking at the plant extracts as anthelmintic, we defined the selectivity index (SI) (Table 4). In this study, the SI of the stem bark is 1.26 for the Chang liver cell (normal cell line) and 8.01 for the HepG2 (cancer line) while it is less than 1 for the root on both cell lines (Table 4).

Considering the anticancer effects of the extracts, the SI with regard to the normal cell line showed to be 0.16 and 1.56, respectively, for stem bark and roots. Thus the stem bark seems to be very less toxic to the cancer liver cell lines but relatively toxic to the normal liver cell line while the roots seem to be more toxic against cancer cell than the normal cell line.

## 4. Discussion

The study of medicinal plants as a new approach for the experimental treatment of schistosomiasis is one of the viable and promising research leads. World Health Organization encouraged the research on medicinal plants by considering that certain traditional knowledge on curative plants could add up to the development of new pharmaceutical products especially to combat diseases that affect populations of underdeveloped countries [32]. Literature searches revealed that many plants have been used to treat schistosomiasis in many African cultures [11, 33–35]. In Mali an ethnopharmacological survey reported fifty-five plants belonging to 30 families to be used for treating urinary and intestinal schistosomiasis, while nine combinations of plants were reported to be used against the urinary form of the disease [34].* Cissus quadrangularis* and* Stylosanthes erecta* were reported as the plants most frequently used against schistosomiasis in Mali [34]. Mølgaard et al. (2001) reported that 21 Zimbabwean plants, traditionally used against schistosomiasis possess activity against schistosomula worms, with* Euclea natalensis* having the highest activity [11, 35]. The results obtained on Zimbabwean plants used to treat schistosomiasis suggested that extracts made from* Abrus precatorius*,* Pterocarpus angolensis,* and* Ozoroa insignis* were lethal to adult schistosomes in vitro [33]. Other plants have been shown to possess different degree of activity against cercariae such as* Glinus lotoides* [36],* Balanites aegyptiaca* [37],* Combretum aculeatum*,* Combretum hartmannianum, Combretum glutinosum,* and* Terminalia laxiflora* [38]. In this study, the promising antischistosomal activity of* R. vomitoria* has been reported for the first time by in vitro tests using alcoholic crude extracts of stem bark and root on* S. mansoni *cercariae and adult worms.

Infections with schistosomes occur when cercariae penetrate and enter into the body through intact skin. Therefore, preventing such penetration of cercariae into the skin is also a potential form of transmission control [6]. Over the years, many topical agents have been evaluated for their ability to block penetration of cercariae into the skin and some of these compounds are highly effective but have not been used clinically due to their potential toxicity and difficulty in formulation and/or applying under field conditions [39]. These among other factors have stimulated the search for new agents that are safer and capable of providing such protection. In this context, one of the first aspects to be analyzed is to evaluate the cercaricidal activity of the potential compounds. In this study the results obtained against cercariae of* S. mansoni* showed that the extracts from* R. vomitoria* (stem bark and root) are active at concentrations below 1000 *μ*g/mL. Extract from the stem bark was active at concentrations from 31.25 *μ*g/mL, while extract from the root was active at higher concentrations from 125 to 1000 *μ*g/mL, and this activity was both time and dose dependent. The present observation showed that ethanol extracts of* R. vomitoria* stem bark and roots possess cercariacidal activity against* S. mansoni* cercariae. This result is similar to that of Kiros et al. [36] and Mohamed et al. [40] who reported the cercariacidal activity of* Glinus lotoides* fruits and* Nigella sativa* crushed seed, respectively, as both time and concentration dependent. Other studies have also reported the time concentration relationship of various plant extracts:* Millettia thonningii* [41],* Iris germanica* [42],* Jatropha curcas* [43], and* Solanum nigrum* [44]. Our results showed that the stem bark cercariacidal activity was more potent than that of the roots and it also had good selectivity in HepG2 cells. The cercariacidal activity of* R. vomitoria* might be due to the presence of flavonoids and other compounds in the plant. Flavonoids isolated from* Millettia thonningii* have been reported to exhibit in vitro cercaricidal activity.

Both plant parts produced 100% mortality after 60 min of exposure at concentration less than 1000 *μ*g/mL (10^−3 ^g/mL). This is far higher than those reported by Ajayi et al. [45] for the species* Tetrapleura tetraptera* (fruits) and* Lagenaria brevifolia* (seeds) which produced 100% mortality after 60 min of exposure at concentrations of 1000 g/mL and 250 mg/mL, respectively.

The viability of adult worms was affected at all the tested concentration (250, 500, and 1000 *μ*g/mL). Thus, there is the possibility of* R. vomitoria* having varying effectiveness against different phases of the life cycle of the parasite.

In this study, the cercaricidal activity shown by the plant extracts was higher than or equivalent to schistosomicidal activity. These results may suggest that the cercariae might be more susceptible than the adult worms. Further studies on mechanism of action would however be needed to understand this feature. The cercariae are covered by a trilaminate layer that protects the inside from any external agent acting on it [46]. Immediately after penetration into the vertebrate host, the resulting schistosomulum is protected by a heptalaminate membrane, which makes it less susceptible than the cercariae [47].

*R. vomitoria* extracts caused motor activity reduction and the death of adult* S. mansoni* worms after 120 h of in vitro exposure. At the same concentration, both extracts also caused the separation of all paired worms and disruption of their tegument. The varying potencies of this plant's components may be due to the different types of compounds present in them. Previous studies have identified the presence of phytochemical such as alkaloids, tannins, saponins, flavonoids, steroids, terpenoids, and cardiac glycosides in the ethanolic extract of* R. vomitoria* [48] and various alkaloids were also isolated and characterized [18, 49].

Terpenoids are known as the largest naturally occurring family of hydrocarbons with a very broad range of biological activities, including antimalarial [50] and anticancer [51] properties and schistosomicidal activity. Some terpenoids are known to kill adult* S. mansoni* worms and cause complete separation of paired worms with tegumental disruption in worms [52]. Several alkaloids such as piplartine [8], piperamide, epiisopiloturine, sanguinarine, solamargine and solasonine [52], mefloquine [53, 54], and artemisinins [55, 56] have been shown to display a broad range of promising biological activities including schistosomicidal property. Many alkaloids display their activities either by causing disruption of tegument, such as sloughing, or by decreasing motor activity and the death of* S. mansoni* adult worms after exposure in vitro [57]. Also they can cause a reduction in egg production and separation of all paired worms [57]. As for flavonoids, they are not able to kill the worms, but they can exhibit significant reduction in motor activity or pairing of the* S. mansoni* adult worms [57].

It is important to establish that a compound or drug candidate under investigation has antischistosomal activity at concentrations that can be achieved in vivo without inducing toxic effects to host cells. In the present study,* R. vomitoria* was evaluated for its cytotoxicity on Chang liver (normal liver) and HepG2 (human hepatocarcinoma) cells using MTT assay. This study made use of complementary approach of whole-organism phenotypic screening in vitro to measure* R. vomitoria* extracts efficacy in order to discover potential anthelmintic candidates for in vivo tests. The efficacy of these plant extracts against schistosomes was defined as curative, by killing the adult worm, and prophylactic, by killing cercariae. Several parameters, such as motor activity, tegumental changes, and death, were evaluated as indicators of schistosomicidal biological activity against the parasites. On the other hand, cytotoxicity tests use a series of increasing concentrations of the antischistosomal product to determine what concentration results in the death of 50 percent of the host cells. This value is referred to as the median cellular cytotoxicity concentration and is identified by IC_50_. The ability of the cells to survive a toxic insult has been the basis of most cytotoxicity assays. Against Chang liver cell, which is the normal liver cell lines, the stem bark and root showed IC_50_ values of 77.5 *μ*g/mL and 112 *μ*g/mL, respectively. These results suggest that the stem bark is relatively more toxic than the roots to the Chang liver cell. For the HepG2, which is a human liver cancer cell, the IC_50_ values were 490.7 and 72.68, respectively, for the stem bark and roots. This means the roots are more toxic to the liver cancer cell lines than the stem bark. Based on the above observations, stem bark is less active against the cancer cell line. In the US NCI plant screening program, a crude extract is generally considered to have in vitro cytotoxic activity, if the IC_50_ value following incubation between 48 h and 72 h is less than 20 *μ*g/mL [58]. Based on this criterion, all the IC_50_ values defined in this study are far higher than 20 *μ*g/mL which indicate that both plant parts are not strongly cytotoxic.

The relative effectiveness of the drug candidate in inhibiting parasite growth compared to inducing cell death is defined as the therapeutic or selectivity index. The selectivity index of* R. vomitoria* stem bark was 1.26 for Chang liver cell lines and 8.01 against HepG2. Based on the SI, the activity of the stem bark is relatively effective against* Schistosoma* parasites. For the roots, the SI were 0.25 and 0.16 against Chang liver and HepG2 cell lines, respectively (<1). Although the roots are active against* Schistosoma* parasites and liver cancer cells (HepG2), the activity is not specific, and they are also toxic to the normal cell lines (Chang liver cell). It is desirable to have a high therapeutic index (>2) giving maximum anthelmintic activity with minimal toxicity on the normal cell. The stem bark of* R. vomitoria* in this study might be more active and specific against schistosome parasites.

Obviously, many variables influence bioactivity both positively and negatively. In vitro testing alone does not necessarily confirm a plant's bioactivity in vivo due to many factors. Furthermore, an extract lacking in vitro activity may still possess in vivo activity since it may be acting as a prodrug. Ideally, a plant extract should be tested both in vitro and in vivo to confirm antischistosomal activity and toxicity. It is therefore recommended that further studies may be conducted on* R. vomitoria* stem bark and roots.

## 5. Conclusions

From the present study, it can be concluded that ethanol crude extracts of* R. vomitoria *stem bark and root possess moderate antischistosomal properties against two life stages of* S. mansoni*: cercariae and adult worms. The two plant parts showed different activity with respect to both time and concentration with the stem bark being more active and specific for schistosome parasites. Since in vitro assays alone are not suitable to cover all aspects of the anthelminthic activities of drugs, especially with respect to pharmacological and immunological host interactions, this study provides first evidence of anthelminthic effects of* R. vomitoria* and this may lead to the consideration of this plant as potential source for new antischistosomal drug. Therefore, further investigation is warranted to evaluate fractions and identify the chemical constituents that elicit activity against* S. mansoni*.

## Figures and Tables

**Table 1:** In vitro cercariacidal effect of ethanol extract of *Rauwolfia vomitoria* stem bark and root on *S. mansoni* cercariae.

Plant parts	Lethal concentrations (*µ*g/mL)		Negative control (% mortality)
	LC_50_		
	1 hour	2 hours	
Stem bark	207.4 CI (141.1 to 306.7)	61.18 CI (55.02 to 68.25)	0
Roots	1430 CI (515.2 to 15271)	452.7 CI (224.9 to 1915)	0

**Table 2:** In vitro effects of crude ethanolic extract of *R. vomitoria* roots and stem bark on the viability of *Schistosoma mansoni* adult worms.

Group	Incubation period (h)	Couples separated worms (%)	Dead worms (%)	Motor activity reduction (%)		Number of worms with tegumental alteration (%)	
				Slight	Significant	Partial	Extensive
Control^a^	2	0	0	0	0	0	0
	24	0	0	0	0	0	0
	48	0	0	0	0	0	0
	72	0	0	0	0	0	0
	96	0	0	0	0	0	0
	120	0	0	0	0	0	0

1% DMSO	2	0	0	0	0	0	0
	24	0	0	0	0	0	0
	48	0	0	0	0	0	0
	72	0	0	0	0	0	0
	96	0	0	0	0	0	0
	120	0	0	0	0	0	0

PZQ^b^	2	0	0	100	0	80	0
	24	0	100	0	100	0	100
	48	40	100	0	100	0	100
	72	40	100	0	100	0	100
	96	100	100	0	100	0	100
	120	100	100	0	100	0	100

*R. vomitoria *stem bark^c^	2	0	0	0	0	0	0
	24	20	0	100	0	40	0
	48	20	0	100	0	80	0
	72	60	0	100	0	80	0
	96	100	0	80	0	80	0
	120	100	100	0	100	80	20

*R. vomitoria* roots^c^	2	0	0	0	0	0	0
	24	40	0	20	80	40	0
	48	40	0	20	80	40	0
	72	40	0	0	100	80	0
	96	100	0	0	100	80	0
	120	100	100	0	100	80	0

^a^RPMI 1640.

^b^Tested at concentration of 10 *μ*g/mL.

^c^Tested at concentration of 250 *μ*g/mL.

**Table 3:** Percentage viability of cell proliferation.

Conc. (*µ*g/mL)	*R. vomitoria* stem bark		*R. vomitoria* root	
	HepG2	Chang Liver	HepG2	Chang liver
	Mean ± SEM	Mean ± SEM	Mean ± SEM	Mean ± SEM
0	100 ± 0	100 ± 0	100 ± 0	100 ± 0
62.5	100.535 ± 0.082	51.716 ± 2.208	73.149 ± 3.892	102.276 ± 0.625
125	86.411 ± 0.048	45.242 ± 1.562	2.662 ± 0.334	31.807 ± 1.585
250	78.841 ± 0.023	20.779 ± 3.644	−0.295 ± 0.082	18.462 ± 0.773
500	51.607 ± 0.119	9.527 ± 1.044	−4.926 ± 0.433	7.523 ± 1.595
1000	18.408 ± 0.251	10.335 ± 1.176	−16.317 ± 2.070	4.025 ± 4.396

**Table 4:** Cytotoxicity of the ethanolic extracts of *R. vomitoria* stem bark and roots.

Cell type	Cell line	IC_50_ (*µ*g/mL), 72 h		SI	
		Stem bark	Roots	Stem bark	Roots
Normal liver	Chang Liver	77.52	112	1.26	0.25
Hepatocarcinoma	HepG2	490.7	72.68	8.01	0.16
Normal/hepatocarcinoma	Chang/HepG2	/	/	0.16	1.54

## Abbreviations

ECACC: European Collection of Authenticated Cell Cultures
ATCC: American Type Culture Collection
DMEM: Dulbecco's Modified Eagle's Medium
DMSO: Dimethyl sulfoxide
DR: Dose response
FBS: Fetal bovine serum
HBSS: Hank's balance salt solution
MTT: 3-(4,5-Dimethyl thiazol-2-yl)-5-diphenyl tetrazolium bromide
MEC: Minimal effective concentration
MLC: Minimal lethal concentration
NIACUC: NMIMR Institutional Animal Care and Use Committee
NMIMR: Noguchi Memorial Institute for Medical Research
NTS: Newly transformed schistosomula
PBS: Phosphate Buffered Saline
PDD: Plant Development Department
PZQ: Praziquantel
RPMI: Roswell Park Memorial Institute
SI: Selectivity index
TDR: Research and Training in Tropical Diseases
UCSF: University of California San Francisco
US NCI: United States National Cancer Institute
WHO: World Health Organization.
